# Feeding Two Birds With One Seed: Using Fluoxetine for Pre-Menstrual Dysphoric Disorder and Bulimia Nervosa

**DOI:** 10.1192/bjo.2024.684

**Published:** 2024-08-01

**Authors:** Prarthana Poodipedi, Parveen Bains

**Affiliations:** Oxford Health NHS Foundation Trust, Oxford, United Kingdom

## Abstract

**Aims:**

Background

This case study describes the use of fluoxetine for reduction of pre-menstrual dysphoric disorder (PMDD) and bulimia nervosa symptoms. The case report also describes an increase in binge purge symptoms in the pre-menstrual period, along with other mood and cognitive symptoms. This supports a hormonal basis to the exacerbation of eating disorders. Patient consent was obtained prior to the publication of this report.

**Methods:**

Case report

A 41-year-old lady with significant binge purge behaviours and mood disturbance was referred to our eating disorder service. She met the diagnostic criteria for bulimia nervosa after a thorough assessment, along with a component of mood dysregulation. She was prescribed sertraline for depressive symptoms in primary care. The patient described a worsening of mood symptoms, along with cognitive difficulties before the start of her menstrual cycle. After a medical review, we agreed on tracking these symptoms along with the binge-purge frequency for a period of two cycles. This was done using a PMDD tracker. The tracker reflected a clear diagnosis of PMDD along with an exacerbation of bingeing and purging symptoms before the start of a menstrual cycle. Following this, sertraline was switched to fluoxetine, and titrated up to its maximum dose of 60 mg a day.

**Results:**

Discussion

Following the commencement of fluoxetine, purging frequency dramatically reduced and subsequently stopped. Although mood symptoms still persisted, the specific mood symptoms along with cognitive symptoms in the pre-menstrual period reduced.

**Conclusion:**

There is some evidence for the use of fluoxetine use for binge purge symptoms in bulimia nervosa. Fluoxetine has also been used either continuously or in the luteal phase for PMDD. This case report reflects the possible correlation between binge-purge symptoms and PMDD symptoms, and the potential use of fluoxetine for dual symptom reduction. PMDD still remains to be a significantly under-diagnosed condition in women. This case report also signifies the importance of exploring PMDD symptoms in eating disorders.

